# Complete vs Near-Complete Reperfusion After Endovascular Therapy for Anterior-Circulation Large-Core Stroke: Propensity-Matched 90-Day Outcomes and Collateral-Modified Time Effects

**DOI:** 10.7759/cureus.106158

**Published:** 2026-03-30

**Authors:** Cheekatla Priyanka, Akudari Vidyasagar, Bijjiga Siphora Krupalini, Keerthiraj B, Misri Zulkifli

**Affiliations:** 1 Department of Neurology, Kasturba Medical College Hospital, Mangalore, IND; 2 Department of Internal Medicine, Sudeeksha Multispeciality Hospital, Nalgonda, IND; 3 Department of Neurology, Aster Medcity, Kochi, IND; 4 Department of Radiodiagnosis, Kasturba Medical College Hospital, Mangalore, IND

**Keywords:** collateral circulation, diffusion-weighted imaging, endovascular therapy, large-core stroke, mtici reperfusion grade

## Abstract

Background: In endovascular therapy (EVT)-treated anterior-circulation large-core stroke, the incremental value of complete reperfusion modified thrombolysis in cerebral infarction (mTICI 3) over near-complete reperfusion (mTICI 2b) is unclear, and collateral physiology may modify time-related tissue injury and recovery.

Methods: This retrospective cohort included adults with anterior-circulation large-vessel occlusion, Alberta Stroke Program Early CT Score (ASPECTS) of 3-5, successful reperfusion (mTICI: ≥2b), pretreatment diffusion-weighted imaging (DWI) core volumetry, and 90-day outcome data (n = 106). A 1:1 propensity score match compared final mTICI 2b versus final mTICI 3 (matched n = 84; 42 per group). Prespecified interaction models tested with American Society of Interventional and Therapeutic Neuroradiology/Society of Interventional Radiology (ASITN/SIR) collateral grade as an effect modifier of onset-to-reperfusion time.

Results: The mTICI 2b group required more thrombectomy passes (median: 2 vs 1; p < 0.001). Functional independence (modified Rankin Scale (mRS): 0 to 2) was similar for mTICI 2b versus mTICI 3 (10/42 (23.8%) vs 11/42 (26.2%); adjusted odds ratio (OR): 1.21; p = 0.66). Symptomatic intracranial hemorrhage (sICH) was higher with mTICI 2b (6/42 (14.3%) vs 2/42 (4.8%); p = 0.04), while 90-day mortality was similar (15/42 (35.7%) vs 13/42 (31.0%); p = 0.75). A longer onset-to-reperfusion time was associated with a larger DWI core (+5.8 cc per hour; p = 0.01) and lower odds of functional independence (OR: 0.78 per hour; p = 0.04). Higher collateral grade was associated with smaller core (-8.4 cc per grade; p = 0.02) and higher odds of independence (OR: 2.5 per grade; p = 0.01). Time × collateral interactions were significant for core (β -2.1; p = 0.03) and independence (OR: 1.19; p = 0.04).

Conclusions: In large-core endovascular therapy (EVT) with successful reperfusion, mTICI 3 did not improve 90-day functional outcomes versus mTICI 2b but was associated with fewer symptomatic hemorrhages, supporting physiology-informed urgency and counselling in this high-risk population. Procedural escalation beyond mTICI 2b should be individualized in large cores overall.

## Introduction

Acute ischemic stroke from anterior-circulation large-vessel occlusion remains a leading cause of death and long-term disability, and endovascular therapy (EVT) has become the cornerstone reperfusion strategy because it improves 90-day disability distributions when added to the best medical management [1-3]. Even with contemporary techniques and high angiographic success rates, outcomes remain heterogeneous, particularly in patients with extensive baseline infarction, where tissue injury may already be advanced and hemorrhagic complications are more frequent. This clinical tension has catalyzed a shift from rigid time-only frameworks toward physiology-informed selection anchored to infarct burden and collateral adequacy [4,5].

Large-core or low-Alberta Stroke Program Early CT Score (ASPECTS) strokes were historically excluded from pivotal EVT trials, yet newer randomized evidence and pooled syntheses indicate that EVT can still yield functional benefit in carefully selected patients with ASPECTS 3-5, albeit with higher risks of intracranial hemorrhage compared with medical therapy alone [6-8]. Observational data further suggest that the benefit may be most consistent when core size is not extreme, while hemorrhagic transformation and futile recovery become more common as infarct burden increases [9]. Earlier registry experience in very low DWI-ASPECTS cohorts also underscored that clinical recovery is tightly linked to achieving meaningful reperfusion rather than partial flow restoration, supporting continued refinement of reperfusion targets in the large-infarct phenotype [10].

Within EVT practice, successful reperfusion is commonly defined as modified thrombolysis in cerebral infarction (mTICI) 2b or better, but the prognostic separation between near-complete reperfusion (mTICI 2b) and complete reperfusion (mTICI 3) is not uniform across studies and may depend on baseline tissue and hemodynamic context. Contemporary work using expanded reperfusion metrics has shown that achieving more complete reperfusion (expanded thrombolysis in cerebral infarction; eTICI 2c/3 versus 2b) is associated with higher rates of good 90-day outcome, while also demonstrating that the incremental benefit of “more complete” angiographic results can be attenuated when tissue-level collaterals are strong and amplified when collaterals are poor [11]. However, dedicated evidence focusing specifically on the mTICI 2b versus mTICI 3 contrast in patients with established large baseline infarction remains limited, even though this subgroup is central to current debates about procedural aggressiveness, pass escalation, and safety trade-offs.

Time from symptom onset to reperfusion remains biologically important, but its impact on infarct evolution is nonlinear across patients because collateral circulation modulates the pace of irreversible injury. Robust collaterals can sustain penumbral perfusion, slow core expansion, and extend the effective therapeutic window, whereas poor collaterals accelerate infarct growth and increase vulnerability to hemorrhagic transformation [4]. Large registry data also indicate that collateral status is independently associated with better 90-day outcomes after EVT, and that the detrimental association of longer onset-to-reperfusion time is steeper when collaterals are poor, implying clinically relevant interaction rather than simple additivity [12]. These concepts align with guideline frameworks that prioritize tissue viability and imaging-derived risk stratification, especially as EVT indications expand into later windows and larger infarct burdens [5,8].

Magnetic resonance diffusion-weighted imaging (DWI) provides sensitive early tissue characterization and allows volumetric estimation of established infarct core, which is strongly linked to post-EVT prognosis and can support more nuanced counselling than clock time alone. Recent clinical cohorts using magnetic resonance imaging (MRI)-based core quantification have shown that smaller baseline DWI core volumes are associated with a higher probability of 90-day functional independence, while symptomatic hemorrhage remains a major determinant of poor outcome [13]. Beyond baseline imaging, infarct growth and early post-procedural DWI lesion volume further refine outcome prediction, reinforcing the relevance of tissue trajectories alongside angiographic endpoints [11,12].

Against this background, the present study was designed to address two practical, interlinked gaps in large-infarct anterior-circulation EVT. First, it compares 90-day functional outcomes and safety between near-complete (final mTICI 2b) and complete (final mTICI 3) reperfusion among patients with large baseline infarction defined by ASPECTS 3-5, using propensity score matching to reduce confounding. Second, it tests whether baseline angiographic collateral grade modifies the associations of onset-to-reperfusion time with pretreatment DWI infarct core volume and with 90-day functional independence, probing whether collateral physiology can explain heterogeneity in time-tissue-outcome relationships in this high-risk population.

## Materials and methods

Study design and patient selection

This retrospective observational cohort study included consecutive adult patients with acute ischemic stroke who underwent EVT for anterior-circulation large-vessel occlusion between February 2023 and August 2024 at the Department of Neurology, Kasturba Medical College Hospital, Mangalore, after approval by the Institutional Ethics Committee (IEC No. IECKMCMLR-12/2022/493; dated December 15, 2022). The study evaluated 90-day functional outcomes, infarct core burden, and safety outcomes among patients with large baseline infarctions who achieved successful angiographic reperfusion. Only anterior-circulation strokes were included to maintain consistency in collateral grading and infarct assessment.

Eligible patients were aged 18 years or older and presented with acute ischemic stroke due to an isolated occlusion of the intracranial internal carotid artery or the M1 or proximal M2 segment of the middle cerebral artery. Large baseline infarction was defined as an ASPECTS [14] of 3 to 5 on pretreatment noncontrast computed tomography (CT) or diffusion-weighted MRI. Additional inclusion criteria were prestroke modified Rankin Scale (mRS) of 0 to 2 [15], admission National Institutes of Health Stroke Scale (NIHSS) score of 24 or less [16], successful reperfusion after EVT (final mTICI; grade at least 2b) [17], availability of pretreatment DWI for infarct core volumetry, and availability of 90-day functional outcome data. Patients with extracranial internal carotid artery occlusion, tandem lesions, posterior-circulation stroke, acute intracranial stenting, or incomplete imaging or outcome data were excluded. A total of 106 patients fulfilled all eligibility criteria and comprised the final study cohort.

Clinical imaging and procedural assessment

Baseline demographic and clinical variables, including age, sex, vascular risk factors, prestroke functional status, admission NIHSS score [16], and use of intravenous thrombolysis, were extracted from institutional stroke registries. Baseline infarct extent was assessed using ASPECTS on pretreatment noncontrast CT or pretreatment MRI [14].

Collateral circulation was evaluated on baseline angiography using the American Society of Interventional and Therapeutic Neuroradiology/Society of Interventional Radiology (ASITN/SIR) collateral grading scale, ranging from 0 (no collaterals) to 4 (complete and rapid collateralization) [17,18]. Infarct core volume was quantified on pretreatment DWI using a semi-automated segmentation workflow with predefined manual correction rules. Angiographic reperfusion was graded using the mTICI scale [17].

Procedural variables included the number of thrombectomy passes, puncture-to-reperfusion time, and imaging-to-reperfusion time. Symptomatic intracranial hemorrhage (sICH) was defined as any intracranial hemorrhage within 24 hours associated with neurological deterioration of 4 or more points on the NIHSS [16].

Outcomes

The primary outcome was 90-day functional status measured by the mRS [15]. Secondary outcomes included independent ambulation (mRS: 0 to 3 at 90 days), functional independence (mRS: 0 to 2 at 90 days), sICH within 24 hours, and all-cause mortality at 90 days.

Statistical analysis

Propensity score matching (1:1, without replacement) was performed to compare patients with final mTICI 2b versus mTICI 3 reperfusion. Propensity scores were generated using multivariable logistic regression, including age, sex, admission NIHSS, baseline ASPECTS, prestroke mRS, and onset-to-admission time, and post-match balance was assessed using standardized mean differences. Continuous and ordinal variables were compared using the Mann-Whitney U test, and categorical variables using the Pearson chi-square test. Median 90-day mRS was compared using the Mann-Whitney U test, while binary outcomes were analyzed using multivariable logistic regression. Interaction analyses assessed collateral grade as an effect modifier of the association between onset-to-reperfusion time and outcomes. Pretreatment DWI core volume was analyzed using multivariable linear regression, and 90-day functional independence using multivariable logistic regression, each with a time × collateral interaction term. A two-sided p-value of <0.05 was considered statistically significant. All analyses were performed using R version 4.2.2 (R Foundation for Statistical Computing, Vienna, Austria).

## Results

Study cohort and baseline characteristics

Among 512 screened patients, 106 met all inclusion criteria and were included in the analysis. After propensity score matching, 84 patients remained in the matched cohort, comprising 42 patients with final mTICI 2b reperfusion and 42 with final mTICI 3 reperfusion (Table 1).

**Table 1:** Baseline characteristics of the propensity-matched cohort (n = 84) NIHSS: National Institutes of Health Stroke Scale; ASPECTS: Alberta Stroke Program Early CT Score; IQR: interquartile range; SD: standard deviation Continuous and ordinal variables were compared using the Mann-Whitney U test. Categorical variables were compared using the Pearson chi-square test

Variable	mTICI 2b (n = 42)	mTICI 3 (n = 42)	Test statistic	p-value
Age, median (IQR), years	73 (62-81)	71 (60-79)	U = 944	0.58
Male sex, n (%)	23 (54.8)	22 (52.4)	χ² = 0.048	0.82
Admission NIHSS, median (IQR)	18 (14-20)	17 (13-19)	U = 968	0.44
Baseline ASPECTS, median (IQR)	4 (3-5)	4 (3-5)	U = 895	0.91
IV thrombolysis, n (%)	13 (31.0)	13 (31.0)	χ² = 0.000	1.00
Collateral grade, median (IQR)	2.6 ± 0.9	2.8 ± 0.8	U = 773	0.33

Procedural characteristics

Procedural complexity differed between reperfusion groups. Patients with mTICI 2b required more thrombectomy passes than those with mTICI 3, while time-based workflow metrics were similar (Table 2).

**Table 2:** Procedural characteristics IQR: interquartile range Procedural variables are presented as median (IQR) and were compared using the Mann-Whitney U test

Variable	mTICI 2b (n = 42)	mTICI 3 (n = 42)	Test statistic	p-value
Thrombectomy passes, median (IQR)	2 (2-3)	1 (1-2)	U = 1250	<0.001
Puncture-to-reperfusion time, median (IQR), min	52 (38-72)	46 (34-65)	U = 1000	0.29
Imaging-to-reperfusion time, median (IQR), min	95 (72-118)	89 (68-112)	U = 974	0.41

Functional outcomes

At 90 days, the median mRS score was 4 (IQR 3-6) in the mTICI 2b group and 4 (IQR 2-5) in the mTICI 3 group, with no significant difference between groups. Rates of independent ambulation and functional independence were also comparable (Table 3).

**Table 3:** Functional outcomes at 90 days IQR: interquartile range; mRS: modified Rankin Scale Median 90-day mRS was compared between groups using the Mann-Whitney U test

Outcome	mTICI 2b (n = 42)	mTICI 3 (n = 42)	Statistical test/model	Test statistic	OR (95% CI)	p-value
Median mRS (IQR)	4 (3-6)	4 (2-5)	Mann-Whitney U test	U = 933	-	0.65
Independent ambulation (mRS: 0-3), n (%)	17 (40.5)	18 (42.9)	Multivariable logistic regression	-	1.09 (0.49-2.41)	0.83
Functional independence (mRS: 0-2), n (%)	10 (23.8)	11 (26.2)	Multivariable logistic regression	-	1.21 (0.50-2.91)	0.66

Collateral status, time to reperfusion, and infarct core

Longer onset-to-reperfusion time was independently associated with larger pretreatment DWI core volumes, while higher collateral grade was associated with smaller infarct cores. A statistically significant time-by-collateral interaction indicated attenuation of time-associated increases in core volume among patients with better collateral circulation. Similar interaction effects were observed for 90-day functional independence (Table 4).

**Table 4:** Interaction models for infarct core and functional outcome mRS: modified Rankin Scale; DWI: diffusion-weighted imaging; OR: odds ratio; CI: confidence interval; Pretreatment DWI core volume was analyzed using multivariable linear regression and 90-day functional independence using multivariable logistic regression, each with a time × collateral interaction term

Outcome	Statistical model	Predictor	Effect estimate (95% CI)	p-value
Pretreatment DWI core volume	Multivariable linear regression with an interaction term	Onset-to-reperfusion time (per hour)	β = +5.8 cc (95% CI: +1.39 to +10.21)	0.01
Pretreatment DWI core volume	Multivariable linear regression with an interaction term	Collateral grade (per 1-grade increase)	β = −8.4 cc (95% CI: −15.48 to −1.32)	0.02
Pretreatment DWI core volume	Multivariable linear regression with an interaction term	Time × collateral interaction	β = −2.1 cc per hour per collateral grade (95% CI: −4.00 to −0.20)	0.03
Functional independence at 90 days (mRS: 0-2)	Multivariable logistic regression with an interaction term	Onset-to-reperfusion time (per hour)	OR = 0.78 (95% CI: 0.62 to 0.99)	0.04
Functional independence at 90 days (mRS: 0-2)	Multivariable logistic regression with an interaction term	Collateral grade (per 1-grade increase)	OR = 2.50 (95% CI: 1.24 to 5.02)	0.01
Functional independence at 90 days (mRS: 0-2)	Multivariable logistic regression with an interaction term	Time × collateral interaction	OR = 1.19 per hour per collateral grade (95% CI: 1.01 to 1.40)	0.04

Safety outcomes

Symptomatic intracranial hemorrhage occurred more frequently in the mTICI 2b group than in the mTICI 3 group. Ninety-day mortality did not differ significantly between groups (Table 5).

**Table 5:** Safety outcomes sICH: symptomatic intracranial hemorrhage Safety outcomes are presented as n (%) and were compared using the Pearson chi-square test

Outcome	mTICI 2b (n = 42)	mTICI 3 (n = 42)	Test statistic	p-value
sICH within 24 hours, n (%)	6 (14.3)	2 (4.8)	χ² = 4.22	0.04
90-day mortality, n (%)	15 (35.7)	13 (31.0)	χ² = 0.10	0.75

## Discussion

Key findings of the study

In this propensity score-matched cohort of anterior circulation large-core stroke patients (ASPECTS 3 to 5) who achieved successful angiographic reperfusion, three findings stand out. First, complete reperfusion (final mTICI 3) was not associated with a measurable improvement in 90-day disability distribution compared with near-complete reperfusion (final mTICI 2b), and rates of independent ambulation and functional independence were similarly low in both groups. Second, safety differed despite comparable functional outcomes: sICH occurred more often after final mTICI 2b than after final mTICI 3. Third, collateral physiology materially shaped the time-tissue-outcome pathway. Longer onset-to-reperfusion time independently correlated with larger pretreatment DWI core volume and lower odds of 90-day functional independence, while higher angiographic collateral grade correlated with smaller cores and better functional recovery, with statistically significant time-by-collateral interactions indicating that robust collaterals attenuated the adverse association of time with both infarct core burden and functional outcome.

Comparison with existing literature

The overall functional outcomes in this large-core EVT population align with contemporary syntheses showing that EVT in ASPECTS 3 to 5 improves outcomes compared with medical therapy, but that absolute rates of functional independence remain modest and hemorrhagic risk is higher [6-8]. The observed functional independence rates in the matched cohort (approximately one-quarter) are also consistent with pooled EVT-only estimates in low-ASPECTS populations [7] and with earlier registry experience in very low DWI-ASPECTS cohorts where reperfusion was necessary for recovery but did not guarantee independence [10].

The neutral functional separation between mTICI 2b and mTICI 3 in this specific large-core stratum contrasts with broader anterior circulation literature suggesting incremental clinical benefit with more complete reperfusion when comparing eTICI 2c/3 versus 2b in mixed-core cohorts [2,3]. A key nuance is that many “more complete reperfusion is better” datasets are enriched for smaller cores and higher salvageable tissue fractions, where restoring near-normal capillary perfusion can translate into meaningful penumbral rescue. In contrast, the large-core phenotype constrains the ceiling of recovery, potentially blunting the marginal functional gain of moving from near-complete to complete reperfusion once a critical amount of irreversible injury is already present. This interpretation is compatible with registry observations that treatment benefit and tissue salvage decline as infarct core enlarges, with uniformly poor outcomes in the most extreme core strata [9].

Importantly, the present study’s collateral interaction findings are directionally concordant with large registry data showing that the detrimental association of longer onset-to-reperfusion time is steeper when collaterals are poor, and that collaterals independently predict better functional outcome after EVT [12]. Mechanistically and conceptually, this matches contemporary collateral biology syntheses in which robust collaterals slow core expansion, reduce the pace of irreversible injury, and preserve functional substrate for longer, thereby reshaping how “time” translates into tissue loss and disability [4,19]. The finding that better collaterals attenuate time-associated core enlargement also complements imaging-centered frameworks that argue for physiology-informed triage rather than a purely clock-driven approach, especially as EVT expands into later windows and higher-risk tissue profiles [4,5].

The safety signal deserves specific contextualization. Large-core EVT is consistently associated with a higher hemorrhage risk than smaller-core EVT and than medical therapy alone [6-8]. In the current analysis, symptomatic hemorrhage was significantly more frequent in the mTICI 2b group despite similar workflow times. This pattern is compatible with the broader complication literature linking intracranial hemorrhage after thrombectomy to factors such as baseline injury severity, procedural complexity, and reperfusion-related vascular injury [20,21]. The observed procedural difference, namely more thrombectomy passes in the mTICI 2b group, supports a plausible pathway where repeated device manipulation, endothelial trauma, distal embolization, and longer cumulative ischemia-reperfusion stress increase blood-brain barrier disruption and hemorrhagic transformation risk, even if puncture-to-reperfusion time is not statistically different between groups [20,21].

Deviations from prior work and plausible explanations

A notable deviation is the lack of functional advantage for complete reperfusion (mTICI 3) over near-complete reperfusion (mTICI 2b). Several explanations are plausible.

Tissue Ceiling Effect in Large-Core Stroke

In ASPECTS 3 to 5, a substantial fraction of eloquent cortex and deep structures is already compromised. Even if mTICI 3 restores macrovascular patency more completely, the microvascular “no-reflow” phenomenon, reperfusion injury, and established infarct burden may limit functional recovery, compressing outcome differences between mTICI categories. This aligns with the broader observation that, beyond certain core sizes, outcome distributions converge despite technical success [9,22].

Metric Granularity and Subgroup Specificity

Many studies showing incremental benefit focus on eTICI 2c/3 versus 2b and include a wide spectrum of baseline cores. In contrast, this analysis isolates a high-risk, large-core subgroup and uses mTICI 2b versus mTICI 3, which may be less sensitive to subtler tissue-perfusion gradients that matter most when salvageable penumbra exists [3,11].

Procedural Confounding by Complexity

The mTICI 2b group required more passes, suggesting more resistant thrombus, higher clot burden, complex anatomy, or underlying intracranial atherosclerotic disease. Even after propensity matching on baseline clinical and imaging variables, such unmeasured technical factors could drive both (i) failure to reach mTICI 3 and (ii) higher hemorrhage and poorer recovery, thereby diluting any “pure” effect of final reperfusion grade on 90-day function.

A second important deviation is that collateral grade retained an independent association and showed significant interaction effects with time, whereas some smaller MRI-based cohorts reported that collateral metrics and time did not remain independently predictive after adjustment once core volume, reperfusion, and hemorrhage were accounted for [13]. Differences in population mix, collateral grading approach, and analytic focus likely explain this divergence. The present study is restricted to large-core ASPECTS 3 to 5 and explicitly models interaction, which can reveal effect modification even when main effects attenuate in conventional multivariable models. In addition, pretreatment DWI core was quantified using a semi-automated segmentation workflow rather than geometric approximation, potentially strengthening the time-core linkage and improving the ability to detect collateral moderation [4,13].

This work contributes novel, practice-relevant evidence in two tightly linked areas. First, it directly evaluates the mTICI 2b versus mTICI 3 contrast within a dedicated large-core anterior circulation EVT cohort using propensity score matching, addressing a gap where most reperfusion-quality studies are dominated by smaller cores or mixed populations. Second, it demonstrates, within this high-risk large-core group, that baseline angiographic collaterals significantly modify the association of onset-to-reperfusion time with pretreatment DWI core volume and with 90-day functional independence, providing a mechanistic explanation for heterogeneity in “time is brain” effects that is particularly consequential when baseline infarction is already extensive [4,12,19].

Clinical implications

Reperfusion Targets and Procedural Escalation

The absence of a clear functional separation between final mTICI 2b and mTICI 3 suggests that, in established large-core infarction, the incremental functional benefit of pursuing mTICI 3 after achieving mTICI 2b may be smaller than in smaller-core populations. However, the lower symptomatic hemorrhage rate with mTICI 3 and the association between mTICI 2b and higher pass counts highlight a safety-relevant procedural message: repeated attempts that fail to convert 2b to 3 may incur harm without proportional functional gain. This supports a more individualized escalation strategy in which the decision to pursue additional passes after reaching mTICI 2b integrates baseline infarct burden, collateral quality, procedural difficulty, and evolving safety risk, rather than reflexively chasing angiographic perfection.

Collateral-Informed Urgency and Selection

The interaction results reinforce that workflow delay is not uniformly harmful across patients. Poor collaterals appear to “penalize” each added hour more strongly in terms of core expansion and reduced functional independence, while better collaterals buffer this penalty. Clinically, this supports two complementary actions: (i) aggressive systems optimization to minimize delays for all patients, with particular urgency when collaterals are poor, and (ii) using collateral assessment as part of nuanced prognostication and counseling in large-core EVT, rather than relying on time alone [5,12,19].

Counseling and Expectations

Even with successful reperfusion, large-core patients have substantial residual disability and mortality, consistent with meta-analytic experience [6,7]. Counseling should explicitly integrate baseline infarct burden, collateral status, and hemorrhage risk, emphasizing that technical success is necessary but not sufficient, and that outcomes remain constrained by baseline tissue injury and early complications [10,20].

Strengths, weaknesses, and limitations

Key strengths include the consecutive large-core EVT cohort with clearly defined eligibility criteria, standardized collateral grading using ASITN/SIR, volumetric DWI core measurement, and the use of propensity score matching to reduce baseline confounding when comparing reperfusion grades. The prespecified interaction modeling directly tests a clinically meaningful physiologic hypothesis, rather than assuming that time effects are homogeneous across collateral strata.

Weaknesses include the inherent constraints of an observational, retrospective design and the possibility of residual confounding from unmeasured technical variables such as clot composition, thrombus length, intracranial atherosclerotic disease, device choice, rescue techniques, and operator strategy, all of which can influence both final reperfusion grade and complication risk. In addition, while matching improved comparability, it reduced the effective sample size, limiting power to detect modest functional differences between mTICI categories.

Several limitations should be considered when interpreting these results. Because inclusion required pretreatment DWI core volumetry and available 90-day follow-up, selection bias cannot be excluded, and the final matched cohort may not fully represent all patients undergoing EVT for large-core stroke in routine practice. The study is retrospective and dependent on recorded workflow times, which may introduce measurement error in onset-to-reperfusion metrics. Collateral grading was angiography-based and may vary with technique, injection quality, and rater interpretation; the absence of a reported blinded core laboratory adjudication may permit misclassification. The cohort excludes tandem lesions, extracranial ICA occlusion, acute intracranial stenting, and posterior circulation strokes, which improves internal consistency but limits generalizability. The analysis focuses on pretreatment core volume and 90-day outcomes without incorporating follow-up infarct volume or infarct growth, which are strong imaging predictors and could further clarify mechanisms linking time, collaterals, reperfusion quality, and recovery. Finally, symptomatic hemorrhage was defined using standard clinical criteria, but detailed hemorrhage subtyping and blood pressure management variables were not analyzed, both of which can influence post-EVT hemorrhage risk [21].

## Conclusions

In anterior circulation large-core stroke patients who achieved successful EVT reperfusion, complete reperfusion (final mTICI 3) did not confer a detectable advantage in 90-day functional outcomes over near-complete reperfusion (final mTICI 2b) after propensity score matching, but was associated with lower sICH. Onset-to-reperfusion time and collateral grade showed strong, biologically coherent associations with pretreatment DWI core volume and 90-day functional independence, with significant time-by-collateral interactions indicating that robust collaterals mitigate the adverse impact of delay. These findings support a physiology-informed large-core EVT strategy that integrates collateral-aware urgency, realistic expectations of tissue-limited recovery, and individualized procedural escalation beyond mTICI 2b.
